# Transcriptome Analyses of β-Thalassemia −28(A>G) Mutation Using Isogenic Cell Models Generated by CRISPR/Cas9 and Asymmetric Single-Stranded Oligodeoxynucleotides (assODNs)

**DOI:** 10.3389/fgene.2020.577053

**Published:** 2020-10-08

**Authors:** Jing Li, Ziheng Zhou, Hai-Xi Sun, Wenjie Ouyang, Guoyi Dong, Tianbin Liu, Lei Ge, Xiuqing Zhang, Chao Liu, Ying Gu

**Affiliations:** ^1^BGI Education Center, University of Chinese Academy of Sciences, Shenzhen, China; ^2^BGI-Shenzhen, Shenzhen, China; ^3^China National GeneBank, BGI-Shenzhen, Shenzhen, China; ^4^Institute for Stem cell and Regeneration, Chinese Academy of Sciences, Beijing, China; ^5^Guangdong Provincial Key Laboratory of Human Disease Genomics, Shenzhen Key Laboratory of Genomics, BGI-Shenzhen, Shenzhen, China; ^6^Guangdong Provincial Key Laboratory of Genome Read and Write, BGI-Shenzhen, Shenzhen, China

**Keywords:** β-thalassemia, *HBB* -28(A>G), isogenic cells, K562, CRISPR/Cas9, assODNs, RNA-seq

## Abstract

β-thalassemia, caused by mutations in the human hemoglobin β (*HBB*) gene, is one of the most common genetic diseases in the world. The *HBB* −28(A>G) mutation is one of the five most common mutations in Chinese patients with β-thalassemia. However, few studies have been conducted to understand how this mutation affects the expression of pathogenesis-related genes, including globin genes, due to limited homozygote clinical materials. Therefore, we developed an efficient technique using CRISPR/Cas9 combined with asymmetric single-stranded oligodeoxynucleotides (assODNs) to generate a K562 cell model with *HBB* −28(A>G) named K562^–28(A>G)^. Then, we systematically analyzed the differences between K562^–28(A>G)^ and K562 at the transcriptome level by high-throughput RNA-seq before and after erythroid differentiation. We found that the *HBB* −28(A>G) mutation not only disturbed the transcription of *HBB*, but also decreased the expression of *HBG*, which may further aggravate the thalassemia phenotype and partially explain the more severe clinical outcome of β-thalassemia patients with the *HBB* −28(A>G) mutation. Moreover, we found that the K562^–28(A>G)^ cell line is more sensitive to hypoxia and shows a defective erythrogenic program compared with K562 before differentiation. Importantly, all abovementioned abnormalities in K562^–28(A>G)^ were reversed after correction of this mutation with CRISPR/Cas9 and assODNs, confirming the specificity of these phenotypes. Overall, this is the first time to analyze the effects of the *HBB* −28(A>G) mutation at the whole-transcriptome level based on isogenic cell lines, providing a landscape for further investigation of the mechanism of β-thalassemia with the *HBB* −28(A>G) mutation.

## Introduction

β-thalassemia is one of most common autosomal recessive blood diseases spread worldwide, caused by either point mutations or deletions in the β-globin (*HBB*) gene (de Dreuzy et al., 2016; Mansilla-Soto et al., 2016). Approximately 80–90 million people carried β-thalassemia, and about 300,000 children with severe thalassemia are born every year (Galanello and Origa, 2010; Weatherall et al., 2010; Origa, 2017). Mutations or deletions of β-globin genes result in the reduction of hemoglobin β chain (β-globin), deformation of hemoglobin tetramer, and subsequent lysis of erythrocytes, finally causing oxygen shortage, bone deformity, organ dysfunction, and even organ failure in many parts of the human body (Cao and Galanello, 2010; Liang et al., 2017). As for the thalassemia major patients, life-long blood transfusion and iron chelation treatments are required for survival, but often accompanied by numerous complications, including arrhythmia, congestive heart failure, hypothyroidism, hypoparathyroidism, hypogonadism, diabetes, osteoporosis, liver cirrhosis, and recurrent infections. Thus, thalassemia has threatened millions of people’s lives for decades and is still a major public health issue (Xu et al., 2015; Kumar et al., 2016).

β-thalassemia mutations are prevalent in the southern part of China and Southeast Asia, and *HBB* −28(A>G) mutation is one of the five most common *HBB* mutations carried by β-thalassemia patients in China (Lai et al., 2017; Liang et al., 2017). In the *HBB* −28(A>G) mutation, adenine (A) base located at 28 base pairs upstream from the cap site is mutated to guanine (G), disrupting the binding of transcription factor of ATA box and decreasing the RNA expression of *HBB* (Orkin et al., 1983). Patients with the homozygous or compound heterozygous −28(A>G) mutation may develop severe anemia or intermedia anemia (Orkin et al., 1983; Cao and Galanello, 2010). Although the description of severe thalassemia was first published more than 90 years ago and a considerable amount of work has been reported to refine the understanding of the pathophysiology of thalassemia syndromes in the past 50 years, the cellular and molecular basis of this group of diseases has still not been thoroughly investigated (Taher et al., 2018). In particular, few studies have been conducted on the *HBB* −28(A>G) mutation to understand how this mutation affects gene expression at the transcriptome level although correcting the *HBB* −28(A>G) mutation with the base editing (BE) system has also been reported in human iPS cells and reconstituted embryos (Liang et al., 2017). Without a full understanding of the defects at the molecular level, it will be difficult to comprehensively evaluate the rescue effect after correcting the mutation.

In a recent study, high-throughput RNA sequencing (RNA-seq) was used to compare control samples with patient samples carrying a novel *HBB* mutation (*HBB*: c.51C > T). It shows that hemopoiesis, heme biosynthesis, response to oxidative stress, and other cellular activity pathways were directly or indirectly enriched by differentially expressed genes related to β-thalassemia (Taghavifar et al., 2019), suggesting that genome-wide RNA-seq analysis is a useful approach to understand the mechanism of β-thalassemia with different mutations. However, control samples in this study are allogenic, and different genetic backgrounds and a mixture of short- and long-term effects would prevent a deep understanding of the effect of this mutation. The cell disease models of hemoglobinopathies have also been reported. Induced pluripotent stem cells (iPSCs) were generated by reprogramming patient-derived cells and provided a good model toward the development of iPSC-based therapy (Li et al., 2011). However, differentiation of human iPSCs into mature and β-globin expressing erythrocytes remains a major challenge in the field. A human umbilical cord blood–derived erythroid progenitor (HUDEP) cell line was established and used as a disease model because of its differentiation ability to produce enucleated red blood cells (Kurita et al., 2013; Traxler et al., 2016; Grevet et al., 2018). However, this cell line is derived from primary hematopoietic stem cells with E6/E7 immortalized basophilic erythroblasts, which depends on erythropoietin (EPO) and cytokine stem cell factor (SCF) for survival, so it is expensive to maintain and hard to do gene editing (Couch et al., 2019). Therefore, we chose the K562 cell line to generate the thalassemia disease cell model for its ready availability, cost-effective maintenance, relatively mature differentiation conditions, and high efficiency of gene editing (Dean et al., 1983; Witt et al., 2000; Ma et al., 2013; DeWitt et al., 2016; Shariati et al., 2016; Ghosh et al., 2018).

In order to explore the specific impact of the *HBB* −28(A>G) mutation on erythroid differentiation and how it affects genome-wide gene expression without confounding factors, such as different genetic background in allogenic samples, we used the CRISPR/Cas9 gene-editing system combined with asymmetric single-stranded oligodeoxynucleotides (assODNs) to generate the disease model of isogenic K562 cell lines (Men et al., 2017; Zhang et al., 2017) and then conducted transcriptome analysis by high-throughput RNA sequencing. The mutant cell line K562^–28(A>G)^ showed no detectable *HBB* gene expression, enabling comparative studies in the same genetic background. We found that *HBB* was transcriptionally prevented by the mutation, and the K562^–28(A>G)^ cell line is more sensitive to hypoxia and present a defective erythrogenic program when compared with wild-type K562 before erythroid differentiation by Gene Ontology (GO) and KEGG analysis. Interestingly, *HBG* showed a lower rate of induction in K562^–28(A>G)^ when compared with wild-type K562 after erythroid differentiation. Taken together, our study is the first to analyze the effects of the *HBB* −28(A>G) mutation at the whole-transcriptome level based on isogenic cell lines. The unraveled molecular biomarkers and signaling pathways that affected in K562^–28(A>G)^ cell line may be further investigated to explore the study of the mechanism of β-thalassemia in the future.

## Materials and Methods

### Generation of β-thalassemia cell line with −28(A>G) mutation

#### sgRNA Design and Construction

**FIGURE 1:**
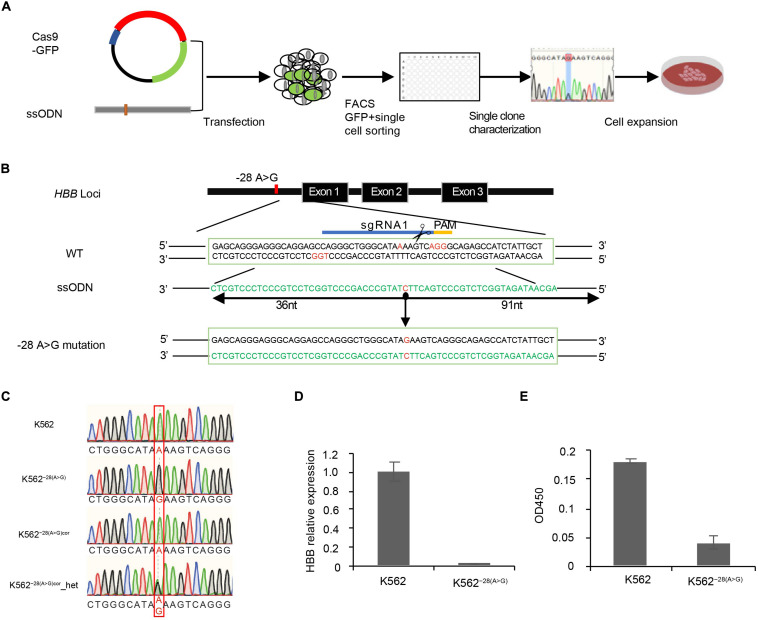
Generation of K562^–28(A>G)^ cell line by CRISPR/Cas9 combined with asymmetric ssODNs. **(A)** Experimental diagram of generation of the *HBB* −28(A>G) mutation cell line in K562. **(B)** The −28 region around HBB is targeted with two sgRNAs and asymmetric ssODNs provided along with CRISPR/Cas9 DNA cleavage to generate −28(A>G) mutation. *sgRNA1* and *sgRNA2* are complementary to the sense and antisense strands, respectively. *sgRNA2* is not labeled but the PAM was showed in red color. Mutation site is indicated with red color in the middle of sequence. PAM: protospacer adjacent motif (orange). **(C)** Identification of −28(A>G; G>A) mutation by sanger sequencing. Expected mutation is shown in the red rectangle. **(D)** Determination of the expression of *HBB* by qRT-PCR in K562^–28(A>G)^ and K562. **(E)** Determination of the expression of *HBB* by ELISA with benzidine staining in K562^–28(A>G)^ and K562.

Two 20-bp sgRNAs were chosen containing the −28(A>G) site and the cutting sites were about 3 and 9 bp before the mutation site, respectively. The sequence shown in Figure 1B. The guiding RNA oligonucleotides were synthesized by BGI and inserted into the gRNA cloning vector pSpCas9(BB)-2A-GFP (PX458) (Add gene 48138) according to the protocol provided by Zhang Feng’s lab (Ran et al., 2013).

#### Design of ss ODNs Repair Templates

The 127-nt asymmetric single-stranded oligodeoxynucleotides (ssODNs) templates were designed by overlapping the CRISPR/Cas9 cleavage site with 36 bp on the PAM-distal side and a 91-bp extension on the PAM-proximal side of the break (Niu et al., 2016; Paquet et al., 2016; Figures 1A,B) and were synthesized by BGI. Once the site of −28(A>G) was mutated by the assODN template, the site for gRNA recognition was blocked, and Cas9 could not cut this site again. It is easily manipulated and seamless on the genome.

#### Gene Editing

To generate the *HBB* −28(A>G; G>A) mutation, 1 × 10^6^ cells were collected and resuspended in 100 μL electroporation solution (Buffer R) at a final density of 1 × 10^7^ cells/mL, and then 3 μg ss ODNs and 5 μg Cas9/sgRNA (Addgene 48138) were added into the 100 μL resuspended cells. Electroporation was completed by the Neon Transfection System (Invitrogen). Electroporated cells were slowly loaded into growth medium without antibiotics and cultured in 5% CO_2_ at 37°C. 48 h after transfection, flow cytometry was used to isolate the single cell into 96-well plates. 1 week later, we characterized the single cell clone by PCR and Sanger sequencing. The resistant colonies were picked and expanded until we generated the expected cell line.

### Cell Culture and Differentiation

K562 cells were obtained from the American Tissue Culture Collection. The cells of K562 and K562^–28(A>G)^ were cultured in glutamine-minus RPMI 1640 medium (Gibco) with 10% FBS in the presence of 1 mM sodium butyrate for 7 days to induce the erythroid differentiation in humidified atmosphere of 5% CO_2_ at 37°C (Shariati et al., 2016). The control cells were cultured in RPMI 1640 medium with 10% FBS and P/S antibiotics for 7 days in humidified atmosphere of 5% CO_2_ at 37°C.

### Quantitative Real-Time PCR (qRT-PCR) Analysis

Total RNA was isolated from the cells by the TRIzol^TM^ Reagent (Invitrogen). Single-stranded cDNA was synthesized with the oligo(dT) primer using the PrimeScript^TM^ RT reagent Kit with gDNA Eraser (Takara), the obtained cDNAs were analyzed by real-time PCR, using the indicated primers. The *HBB* primers were 5′- GCTCGGTGCCTTTAGTGATG -3′ (forward) and 5′- GCACACAGACCAGCACGTT -3′ (reverse); for *HBG*, 5′- GGAAGATGCTGGAGGAGAAACC -3′ (forward) and 5′- GTCAGCACCTTCTTGCCATGTG -3′ (reverse). The cycling conditions were 95°C denaturation for 10 min, 95°C for 15 s, annealing and extension at 60°C for 40 s, 40 cycles on the ABI step one machine.

### Comparison of the Hemoglobin Expression

#### ELISA

To compare the hemoglobin expression of the uninduced cells [K562, K562^–28(A>G)^, K562^−28(A > G)*cor*^] and induced cells [K562-dif, K562^–28(A>G)^-dif], we used the tetramethylbenzidine (TMB) ELISA Kit (Invitrogen): 1 × 10^5^ cells from each of the test and control groups were resuspended in phosphate buffered saline (PBS) and mixed with an equal volume of the TMB solution and then kept at room temperature for 30 minutes. Then add a double volume of TMB stop solution to the wells. Brown-blue wells were regarded as positive and read at 450 nm by Biotek epoch. Each treatment was performed in triplicate, and each experiment was repeated three times.

#### Flow Cytometry

FITC-conjugated CD235ab (BioLegend) was used as the erythroid-specific surface marker. About 5 × 10^5^ cells were harvested, counted, and resuspended in 100 mL PBS with Human BD Fc Block^TM^(BD) for about 10 min at room temperature; then we added 100 mL FITC-conjugated CD235ab antibody diluted by 1:10 and the samples were incubated at 4°C for 30 min. Cells were washed with ice-cold PBS three times, and the fluorescence was measured by flow cytometry of BD Aria III. The unstained group was used as control for each sample [K562, K562^–28(A>G)^, K562-dif, K562^–28(A>G)^-dif].

### RNA-Seq Analysis

There are more than 65 million 100 bp pair-ended reads per sample. Prior to assembly, raw reads were filtered by SOAPnuke (Chen et al., 2017) with the parameters “-l 15 -q 0.2 -n 0.05”. Reads were mapped to the human reference genome using HISAT2 (Kim et al., 2015), which included both the genome sequences (GRCh38.p12) and known reference sequence (RefSeq) transcripts. Finally, 80% of reads were aligned to the human genome.

We used Samtools (Li et al., 2009) to sort and index the alignment files, and the Integrative Genomics Viewer (IGV) tool (Robinson et al., 2011) was used to visualize the reads. The sorted binary sequence analysis file (BAM files) was also used to generate UCSC browser tracks with a genome coverage bed from Bed Tools (Dennis et al., 2003; Quinlan and Hall, 2010). To this end, coverage files were normalized using the total signal for each sample.

The StringTie (Pertea et al., 2015) suite of tools was used to calculate and compare gene expression levels, which were normalized as fragments per kilobase of transcript per million (FPKMs) mapped reads. Differential expression analysis was assessed at each sample by comparing the pre- and post-induction cells using the EdgeR Bioconductor package (Robinson et al., 2010). Differentially expressed genes (DEGs) were identified when the fold change (FC) > 2 and *p* < 0.05.

### Gene Ontology (GO) Analysis

Gene ontology enrichment analysis of the gene sets (Yu et al., 2012) were performed on each sample using the cluster Profiler R package, including KEGG (Kanehisa and Goto, 2000) pathways and Gene Ontology (Ashburner et al., 2000)^1^, which were collected in the molecular signatures database (MSigDB) (Liberzon et al., 2011). Meanwhile, DAVID^2^ and Metascape were utilized to assess the enrichment of functional categories (GO and KEGG) of the DEGs (Dennis et al., 2003; Zhou et al., 2019).

### Transcription Factor Prediction

To compare the expression levels of transcription regulator genes between pre- and post-induction K562 cells, we collected a comprehensive transcription factor annotation from AnimalTFDB 3.0 (Hu et al., 2018) and iRegulon (Janky et al., 2014) and the results were visualized using Cytoscape (Shannon et al., 2003).

## Results

### Generation of *HBB* −28(A>G) Mutant Cell Line by CRISPR/Cas9 and Asymmetric Single-Stranded Oligodeoxynucleotides

To study how the *HBB* −28(A>G) mutation affects gene expression at the genome-wide level, an isogenic human cell line carrying this mutation was generated. The diagram of the generating mutant cell line is shown, including transfection, CRISPR/Cas9 editing, single cell sorting, cell characterization, and expansion (Figure 1A). As previous studies demonstrated that using ssODNs (Niu et al., 2016) or asymmetric double-strand DNA (Paquet et al., 2016) as a repair template resulted in a higher efficiency of accurate replacement of target sequences through homology-directed repair (HDR), we developed a technology that combined CRISPR/Cas9 with asymmetric ssODNs (assODNs). To generate the *HBB* −28(A>G) mutation, sgRNA mediating DNA cleavage 3 bp aside from the −28 mutation site and assODNs with 36 bp on the PAM-distal side and 91 bp on the PAM-proximal side of the cutting site were used (Figure 1B). K562, a human erythroleukemia line that resembles undifferentiated erythrocytes, was transduced with these Cas/sgRNA and assODNs. We identified one clone with the *HBB* −28(A>G) mutation that showed a single peak at the mutation site by sanger sequencing and named it K562^–28(A>G)^ (Figure 1C). In order to exclude the possibility that the single peak of *HBB* −28(A>G) is generated by a large deletion of the other allele, we then performed CRISPR/Cas9-mediated gene correction on this K562^–28(A>G)^ cell clone and obtained heterozygosity on the mutation site in our heterozygous correction clone K562^−28(A > G)*cor*^-het (Figure 1C), suggesting both alleles of *HBB* are present in the original mutant cell clone K562^–28(A>G)^ and, thus, confirming the homozygosity of K562^–28(A>G)^. In order to reconfirm the functional effect of the *HBB* −28(A>G) mutation, we used qRT-PCR and ELISA to detect the expression of *HBB* mRNA and HBB protein, respectively. In agreement with the sequencing result, the expression of both *HBB* mRNA and HBB protein were undetectable in the K562^–28(A>G)^ cell line. In contrast, wild-type K562 shows a considerable expression of *HBB* mRNA and HBB protein even without erythroid differentiation (Figures 1D,E). These results suggest that the mutant cell line of *HBB* −28(A>G) was successfully generated in which the expression of *HBB* was eliminated.

### Transcriptome Analysis of K562^–28(A>*G)*^ and K562 Before Erythroid Differentiation

**FIGURE 2:**
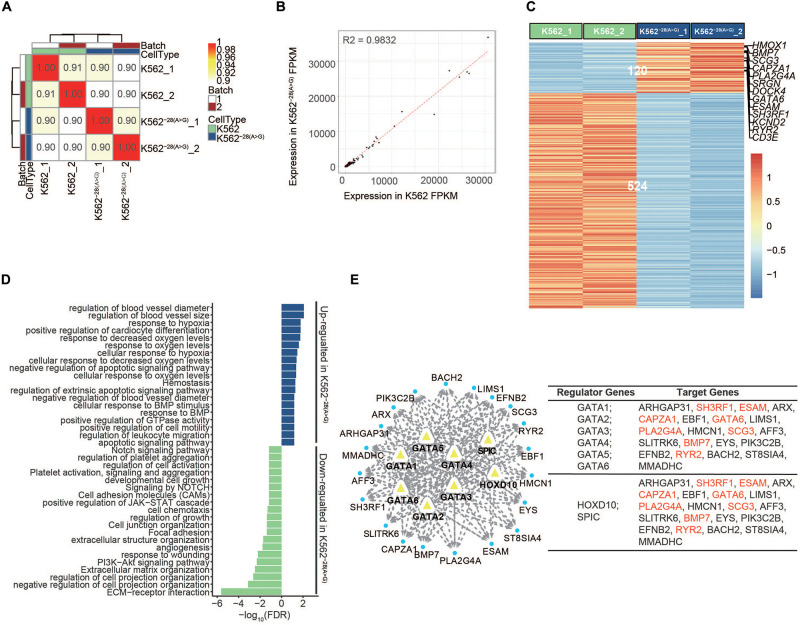
Transcriptome analysis of K562 and K562^–28(A>G)^ cell lines before erythroid differentiation. **(A)** Pairwise Pearson correlations are represented in matrix between K562 and K562^–28(A>G)^ in two batches before differentiation. **(B)** The correlation between K562 and K562^–28(A>G)^ before differentiation. **(C)** Heat map shows the differentially expressed genes (DEGs) between K562 and K562^–28(A>G)^ cell lines; genes related to hypoxia are showed on the right. **(D)** Enrichment analysis of GO, KEGG, and Reactome pathways based on DEGs in K562^–28(A>G)^ before differentiation; the upregulated pathways are shown in blue, and the downregulated pathways are shown in green. **(E)** The predicted interaction network of transcription factors (TFs) in DEGs and their target genes in K562^–28(A>G)^ before differentiation. The hypoxia genes are shown in red.

Many K562^–28(A>G)^ cells appear to have irregular morphology and spontaneous cell death compared to the wild-type counterpart, suggesting loss of *HBB* expression by −28(A>G) mutation may compromise cell viability in normal culture condition (Supplementary Figure S1). To understand how the mutation affects the molecular function of cells at the transcriptional level, we conducted RNA-seq to analyze the transcriptome differences between the isogenic cell line of K562^–28(A>G)^ and its control K562. Pairwise Pearson correlation analysis revealed high similarity between replicates from K562^–28(A>G)^ and K562 cells, indicating high reproducibility of our data (Figure 2A). Overall, the gene expression levels between K562^–28(A>G)^ and K562 cell are similar on a transcriptome level, suggesting that the mutation may not affect the cell identity (Figure 2B). We conducted analysis of differentially expressed genes (DEGs) and found 120 and 524 genes were consistently upregulated and downregulated, respectively, in K562^–28(A>G)^ compared to K562 (Figure 2C). Interestingly, K562^–28(A>G)^ had a higher expression of genes related to the mitochondrial electron transport chain, such as MT-CO1 and MT-ND, implying that the mutant cell line may need more energy and be subject to oxidative stress (Supplementary Table S2). To further explore the affected underlying biological functions, we conducted GO term, KEGG, and Reactome analysis using those DEGs. Interestingly, the PI3K-Akt signaling pathway that is important for erythrocyte differentiation (Jafari et al., 2019) was downregulated in K562^–28(A>G)^ (Figure 2D). Furthermore, we found pathways of (cellular) response to hypoxia and response to (decreased) oxygen level were upregulated in K562^–28(A>G)^ mutant cell line (Figure 2D). Consistently, hypoxia-related genes, such as *HMOX1, BMP7, GATA6, ESAM*, and *RYR2* were upregulated in K562^–28(A>G)^ (Figure 2C). To further explore the core regulators of hypoxia, we performed an interaction assay to predict the interaction of hypoxia-related DEGs and transcription factors (TFs) that target upregulated genes in K562^−28(A > G)^. In agreement with previous results, we observed the hypoxia-related genes, such as *HMOX1* and *SRGN* on the PPI network, and the GATA family, *HOXD10* and *SPIC*, which are well-known regulators of erythroid differentiation and hypoxia (Myers et al., 2002; Bennett et al., 2019) were the core regulators for upregulated genes in K562^–28(A>G)^ (Figure 2E and Supplementary Figure S3). The regulators and their corresponsive target genes were listed, and genes related to the hypoxia response are labeled in red (Figure 2E). Taken together, these data suggest the hypoxia response was upregulated in K562^–28(A>G)^ and the core regulators were the GATA family, HOXD10 and SPIC.

### Transcriptome Analysis of Coregulated Genes in K562^–28(A>G)^ and K562 Cell Lines After Erythroid Differentiation

**FIGURE 3:**
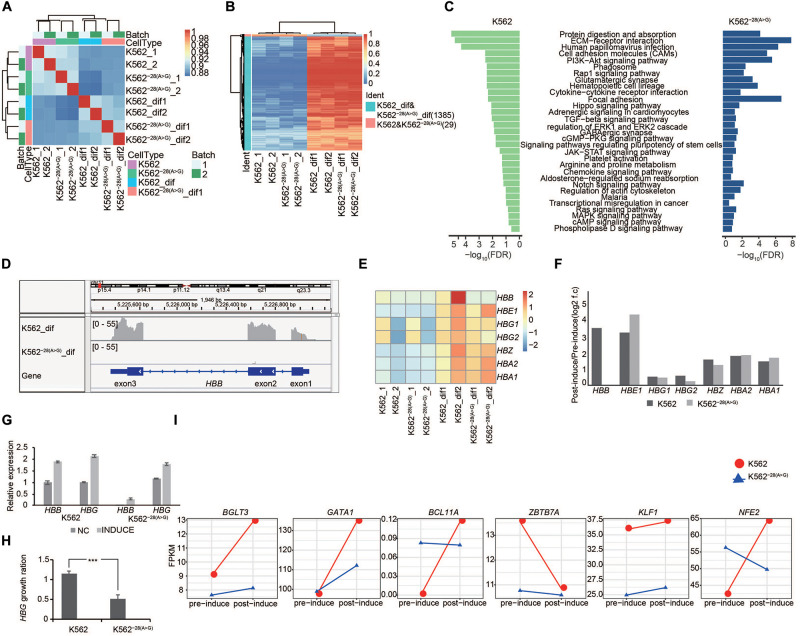
Transcriptome analysis of coregulated genes in K562 and K562^–28(A>G)^ cell lines after erythroid differentiation. **(A)** Pairwise Pearson correlations are represented in matrix between K562 and K562^–28(A>G)^. The differentiated groups are clustered together. **(B)** Heat map shows the coregulated genes in K562 and K562^–28(A>G)^ after erythroid differentiation when compared to those before differentiation. **(C)** KEGG signaling pathways enriched in differentiated K562 and K562^–28(A>G)^ when compared to their corresponding cell lines before differentiation. **(D)** The IGV shows the *HBB* gene expression in K562 and K562^–28(A>G)^ cell lines. **(E)** Expression of globin genes determined by RNA-seq in the K562 and K562^–28(A>G)^ cell lines pre- and post- induction. **(F)** Change trends, post-induction FPKM/pre-induction FPKM (log2 f.c) of globin genes determined by RNA-seq in the K562 and K562^–28(A>G)^ cell lines pre- and post- induction. **(G)** Expression of globin *HBB* and *HBG* determined by qRT-PCR in the K562 and K562^–28(A>G)^ cell lines pre- and post- induction. **(H)** Change trends of globin *HBB* and *HBG* determined by qRT-PCR in the K562 and K562^–28(A>G)^ cell lines pre- and post- induction. *T*-test was used to process statistical analysis. *** means *p* < 0.001. **(I)** Expression of key transcription factors related to erythroid differentiation. Y axis represents expression level (FPKM).

The K562 cell line is often used to study erythroid differentiation *in vitro*. In order to investigate the effect of the *HBB* −28(A>G) mutation during erythroid differentiation, we induced erythroid differentiation using sodium butyrate, confirmed the differentiation efficiency (Supplementary Figure S6), and then performed genome-wide RNA-Seq in the K562 and K562^–28(A>G)^ mutant cell line. Pairwise Pearson correlations represented in the matrix indicated that the isogenic cell lines showed high similarities up to 90%, and the samples were clustered together depending on their differentiation conditions (Figure 3A). The expression of 1,385 genes were consistently upregulated in differentiated K562^–28(A>G)^ and K562 when compared to undifferentiated samples (Figure 3B and Supplementary Figure S4A), which means the mutation couldn’t prevent the process of erythroid differentiation, and these DEGs were used to conduct the GO term, KEGG, and Reactome analysis. Multiple signaling pathways were activated in both K562^–28(A>G)^ and K562 (Figure 3C), including PI3K-Akt, MAPK, and ERK pathways that have been reported to be activated during erythroid differentiation (Witt et al., 2000; Yang et al., 2001; Grass et al., 2003; Jafari et al., 2019). In addition, signal pathways, such as cell adhesion, pluripotency of stem cells, platelet activation, and Notch pathway, were also coactivated in differentiated samples (Figure 3C), suggesting our induction process was successful in both cell lines. We also performed a comparative analysis of erythrocytes pre- and post- induction of K562^–28(A>G)^ and K562. GO analysis showed that erythrocytes take up carbon dioxide and release oxygen pathway and O_2_/CO_2_ exchange in the erythrocytes pathway were enriched in mutant cell lines (Supplementary Figures S4B,C).

*HBB* belongs to the globin gene family, mainly including *HBA* (α-globin), *HBG* (γ-globin), and *HBE* (ε-globin). *HBB, HBG*, and *HBE* are β-globin-like and all capable of forming a tetramer with α-globin. They are located within a gene cluster on chromosome 11, and their expression is coordinated by the same locus control region (LCR) as other regulatory DNA elements (Palstra et al., 2008). In β-thalassemia patients, *HBG* may be upregulated to compensate the loss of *HBB.* To study the compensatory gene expression of globin genes in K562^–28(A>G)^ after differentiation [K562^–28(A>G)^-dif], the expression of *HBB*, *HBA*, and *HBG* was analyzed by RNA-seq in isogenic cell lines of K562^–28(A>G)^ and K562. As expected, IGV analysis showed that *HBB* expression was induced but undetectable in K562^–28(A>G)^ after differentiation (Figure 3D). In contrast, expression of other globin genes was induced and detected in K562^–28(A>G)^ after differentiation (Figure 3E). We noticed that the fold-induction rates of *HBA1, HBA2*, and *HBE1* were similar or slightly increased while *HBZ* slightly decreased in K562^–28(A>G)^-dif when compared to those in K562-dif. However, *HBG* expression in K562^–28(A>G)^-dif was two-fold lower by FPKM than that in K562-dif (Figure 3F). The expression of *HBB* and *HBG* before and after differentiation were further confirmed by qRT-PCR. Consistently, expression of *HBB* was undetectable in undifferentiated K562^–28(A>G)^, and its expression level in K562^–28(A>G)^-dif after differentiation was negligible when compared to K562-dif control (Figure 3G). In agreement with RNA-seq results, the expression level of *HBG* was increased in both cell lines after differentiation, but the induction level of *HBG* caused by differentiation was nearly two-fold lower in K562^–28(A>G)^ than that in K562 (Figures 3G,H). We further analyzed the expression of key TFs that are important for the regulation of globin genes during erythroid differentiation. *KLF1*, a previously reported positive regulator of *HBG* expression (Zhou et al., 2010), is found downregulated in K562^–28(A>G)^. Expression of other positive regulators, including *GATA1*, *BGL3*, and *NFE2* (Grass et al., 2003; Stamatoyannopoulos, 2005; Zhou et al., 2010; Wienert et al., 2018), were dramatically upregulated in K562 after differentiation, and those increases were largely attenuated in K562^–28(A>G)^. In contrast, negative regulators *BCL11A* and *ZBTB7A* were upregulated in K562^–28(A>G)^ compared to K562 before differentiation (Figure 3I and Supplementary Figure S5). The similar expression pattern of globin genes and key translation factors between K562^–28(A>G)^ and K562 suggest the erythroid differentiation is generally normal in K562^–28(A>G)^, but the mutation of *HBB* with −28(A>G) may affect the *HBG* expression through a network of transcription factors.

### Transcriptome Analysis of DEGs in K562 and K562^–28(A>G)^ Cell Lines After Erythroid Differentiation

**FIGURE 4:**
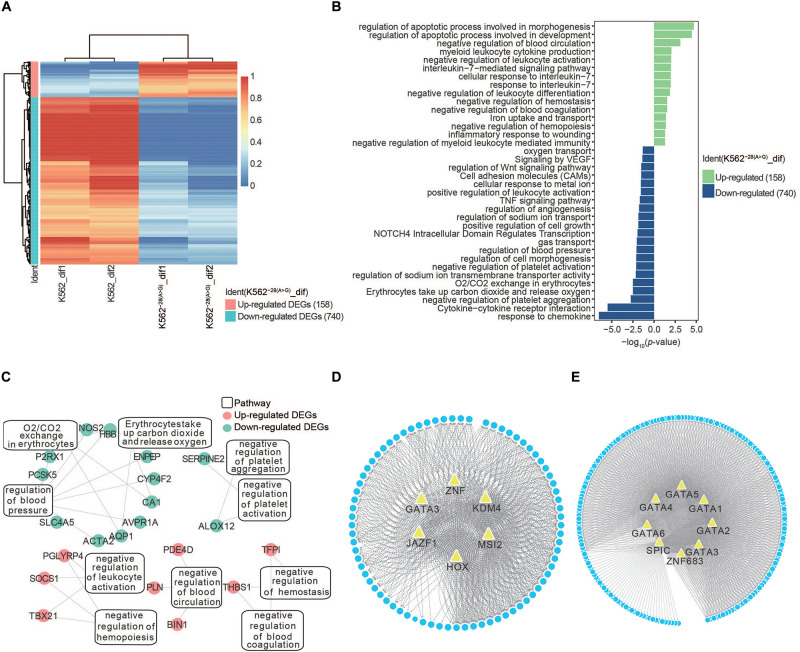
Transcriptome analysis of DEGs in K562 and K562^–28(A>G)^ cell lines after erythroid differentiation. **(A)** Heat map shows DEGs in K562^–28(A>G)^ compared to K562 after differentiation. **(B)** Pathway analysis of K562^–28(A>G)^ compared to K562 after differentiation. The upregulated pathways are shown in green, and the downregulated pathways are shown in blue. **(C)** Enriched pathways and their DEGs in O_2_/CO_2_ exchange in erythrocytes, negative regulation of hemopoiesis, as well as negative regulation of hemostasis. Upregulated DEGs are shown in red, and downregulated DEGs are shown in blue. **(D)** The prediction of TFs in K562 after differentiation. **(E)** The prediction of TFs in K562^–28(A>G)^ after differentiation.

To understand the effect of the *HBB* −28(A>G) mutation during erythroid differentiation, we identified the DEGs (*p* < 0.05, fold change [FC] > 2) within K562^–28(A>G)^-dif and K562-dif. The number of upregulated and downregulated DEGs in K562^–28(A>G)^ were 158 and 740, respectively (Figure 4A). With the GO term, KEGG, and Reactome analysis, we found that upregulated DEGs in K562^–28(A>G)^-dif were enriched in pathways related to stress-response and hematopoiesis disorder, such as regulation of the apoptosis process, negative regulation of leukocyte activation, myeloid leukocyte cytokine production, negative regulation of blood coagulation, negative regulation of hemostasis, and negative regulation of hemopoiesis. Meanwhile, downregulated DEGs in K562^–28(A>G)^-dif were enriched in oxygen-related pathways, including oxygen transport; erythrocytes take up carbon dioxide and release oxygen and O_2_/CO_2_ exchange in erythrocytes (Figure 4B). Pathway analysis of critical DEGs in K562^–28(A>G)^-dif reveal the oxygen transport and blood circulation were enriched (Figure 4C). And *PDE4D*, *TFPI, CA1*, and *AQP1*, which were related to hypoxia, were found to be critical targets (Figure 4C and Supplementary Table S1). Moreover, TF prediction revealed that JAZF1, MSI2, KDM4, HOX family, and ZNF family were core regulators for upregulated genes in K562^–28(A>G)^-dif (Figure 4D) while the SPIC and GATA family are core regulators for downregulated genes in K562^–28(A>G)^-dif (Figure 4E). Interestingly, GATA3 was found to be the core regulator for both upregulated and downregulated genes (Figures 4D,E). Taken together, dysregulated genes and pathways were present in K562^–28(A>G)^ after differentiation and transcription factors, such as GATA, HOX, and ZNF families, may play important roles as core regulators.

### Reversion of Observed Abnormalities in Corrected K562^–28(A>G)*cor*^ Cell Line

**FIGURE 5:**
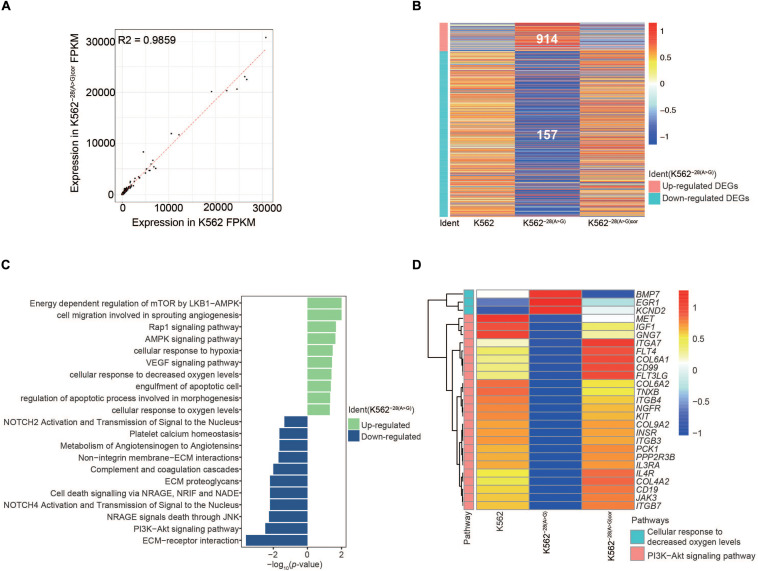
Genes and key pathways are reversed in mutation-corrected K562^−28(A > G)*cor*^ cell line. **(A)** The correlation between K562 and K562^−28(A > G)*cor*^ cell lines. **(B)** The upregulated and downregulated DEGs in K562, K562^−28(A > G)^, and K562^−28(A > G)*cor*^. **(C)** Differentially regulated signaling pathways in K562^–28(A>G)*cor*^ compared to K562^−28(A > G)^. **(D)** The recovery of key pathways.

As it took weeks for the generation of K562 mutant cells by CRISPR/Cas9 and clone selection, it is possible that the differential gene expression is due to secondary effects of the editing process, similar to studies of patient samples (Taghavifar et al., 2019). In order to exclude the potential influence on secondary effects on our transcriptome analyses, we further corrected the mutation in K562^–28(A>G)^ as mentioned before and used a homozygous correction cell line K562^−28(A > G)*cor*^ to perform transcriptome comparison (Figure 1C and Supplementary Figure S2). As expected, we found the expression profile of K562^−28(A > G)*cor*^ had a high relationship with K562 (Figure 5A). Importantly, DEGs heatmap shows a highly similar pattern between K562^−28(A > G)*cor*^ and K562 (Figure 5B), indicating that the editing process by CRISPR/Cas9 did not generate obvious effects on transcriptome. Through analysis of pathway enrichment, the PI3K pathway and cell response to the oxygen pathway were also recovered in K562^−28(A > G)*cor*^ (Figure 5C). The genes of key pathways were also recovered (Figure 5D). In summary, a large portion of abnormalities observed in K562^–28(A>G)^ are reversed in corrected K562^–28(A>*G)*cor**^, suggesting these phenotypes are specifically caused by mutation of *HBB* −28(A>G) and not caused by editing process.

## Discussion

In our study, we successfully generated the cell line K562^–28(A>G)^ of *HBB* with the *–28*(A>G) mutation using CRISPR/Cas9 and a 127 nt assODN. This cell-based disease model was used to study how the *HBB −28*(A>G) mutation affected the cellular function on the transcriptome level before and after erythroid differentiation.

Our results showed the *HBB* −28(A>G) mutation prevented the transcription of *HBB* gene. Analysis of enriched pathways suggested the PI3K pathway as well as the JAK-STAT pathway, which play important roles in erythroid differentiation, were disrupted in K562^–28(A>G)^ before erythroid differentiation. The PI3K-Akt signaling pathway is a significant pathway that controls many cellular processes known as cell division, autophagy, survival, and differentiation (Jafari et al., 2019). Moreover, the mutation activated the hypoxia pathway in undifferentiated K562^–28(A>G)^. Many clinical manifestations observed in β-thalassemia are attributed to the chronic hypoxic environment due to pathologic erythrocyte production, and our data suggest hematopoietic precursors may also be subject to oxidative stress before differentiation.

To induce erythroid differentiation, we chose the glutamine-minus medium with sodium butyrate, as hemoglobin synthesis was markedly induced using this condition with a differentiation efficiency of 11–19% in K562 (Qun and Yuan, 2007; Bruchova-Votavova et al., 2010). Consistent with previous reports, the differentiation efficiency of K562 in our study was nearly 12% (Supplementary Figure S6), indicating that our erythroid differentiation is effective. In agreement, the MAPK and ERK pathway was activated in both K562 and K562^–28(A>G)^ (Figure 2), a finding consistent with observations in previous studies (Witt et al., 2000; Park et al., 2001). Interestingly, the PI3K-Akt signaling pathway was activated in K562^–28(A>G)^ after induction, suggesting the defective PI3K pathway may be caused by a lack of activators in undifferentiated K562^–28(A>G)^. Other pathways, such as cell adhesion, pluripotency of stem cells, platelet activation, and Notch pathway, were also coactivated in differentiated K562 and K562^–28(A>G)^ samples, indicating mutation of *HBB* −28(A>G) did not block the pathways required for differentiation. Nevertheless, consistent with data from undifferentiated K562^–28(A>G)^, oxygen-related pathways were downregulated in differentiated K562^–28(A>G)^ (Figure 4). In both undifferentiated and differentiated conditions, SPIC and GATA families are predicted as core regulators (Figures 2, 4). The GATA family of transcription factors (GATA1–6) are essential for normal hematopoiesis and a multitude of other developmental processes (Grass et al., 2003; Li et al., 2012; Alsayegh et al., 2019; Castano et al., 2019). *GATA-1* regulates terminal differentiation and the function of erythroid, which activates or represses the erythroid-specific gene, such as β-globin locus-binding protein, and it might regulate the switch of fetal to adult hemoglobin in human (Sankaran et al., 2010). Interestingly, increased expression of *GATA1* was largely attenuated in K562^−28(A > G)^ during erythroid differentiation (Figure 3), which may play a role for dysregulated oxygen-related pathways.

As improving the levels of *HBG* in adults could partially reverse the severity of symptoms in sickle disease and β-thalassemia, it is important to understand the coordinated regulation between *HBB* and *HBG* (Chen et al., 2009; Traxler et al., 2016; Li et al., 2018). In this study, we noticed that fold induction of *HBG* was decreased in K562^–28(A>G)^. *ZBTB7A* and *BCL11A* were two major repressors of *HBG* by directly binding the *HBG* gene promoters (Masuda et al., 2016; Liu et al., 2018; Martyn et al., 2018; Wienert et al., 2018). Expression of *ZBTB7A* was decreased during differentiation in K562, consistent with increased expression of *HBG*. However, the overall expression level was higher in K562 than that in K562^–28(A>G)^, paradoxical with the results of decreased fold induction of *HBG* in a mutant cell line. Although expression of *BCL11A* was lower in K562 when compared to K562^–28(A>G)^ before differentiation, its expression increased dramatically during differentiation (Figure 3I and Supplementary Figure S5). Our uncoordinated expression data suggest that the regulation of *HBG* by *ZBTB7A* or *BCL11A* should be a comprehensive process, and the detailed interaction between factors on *HBG* regulation requires further investigation. In summary, our RNA-seq data from isogenic K562 cell models suggest multiple pathways, including PI3K-AKT, JAK-STAT, and oxygen-related pathways, and GATA families may be involved in thalassemia pathogenesis caused by *HBB* −28(A>G) mutation. However, these results may require further validation in clinical samples or alternative models.

Last, we not only used CRISPR/Cas9 to generate mutation, but also corrected the mutation with the same strategy. In one aspect, reversed abnormalities in corrected cell line confirmed the specificities of these phenotypes. In another aspect, the efficient editing results indicate our gene editing strategy using assODNs is powerful and provide means for gene editing treatment of *HBB* −28(A>G) mutation.

In summary, we show the isogenic K562^–28(A>G)^ cell line generated with CRISPR/Cas9 and assODNs is a valuable model to evaluate the β-thalassemia with homozygous mutation of *HBB* with −28(A>G). It provides us with the first transcriptome data for mechanistic study on the effects of *HBB* −28(A>G), and the mutant cell line generated in our study can serve as a cellular disease model to screen lentivirus vectors in potential gene therapies or to evaluate the gene-editing strategies targeting *HBB* −28(A>G) mutation.

## Data Availability Statement

The datasets for this study can be found in the CNSA. Please see the https://db.cngb.org/cnsa/ of CNGBdb with accession code CNP0000981 for more details.

## Author Contributions

YG, CL, and XZ conceived and designed the study. JL performed experiment from LG, TL, GD, and WO. ZZ and H-XS performed bioinformatics analysis. JL, ZZ, YG, and CL wrote the manuscript with the inputs from all authors. All authors contributed to the article and approved the submitted version.

## Conflict of Interest

All authors were employed by BGI-Shenzhen which is a non-profit organization. The authors declare that the research was conducted in the absence of any commercial or financial relationships that could be construed as a potential conflict of interest.

## Footnotes

1 http://www.geneontology.org/

2 https://david.ncifcrf.gov/
