# Supplementary material for: Transcriptome Analyses of β-Thalassemia −28(A>G) Mutation Using Isogenic Cell Models Generated by CRISPR/Cas9 and Asymmetric Single-Stranded Oligodeoxynucleotides (assODNs)
Source: Front Genet. 2020 Oct 8;11:577053. doi: 10.3389/fgene.2020.577053 (PMC7580707; doi:10.3389/fgene.2020.577053)
Supplement: Supplementary file 1 [file Data_Sheet_1.PDF]

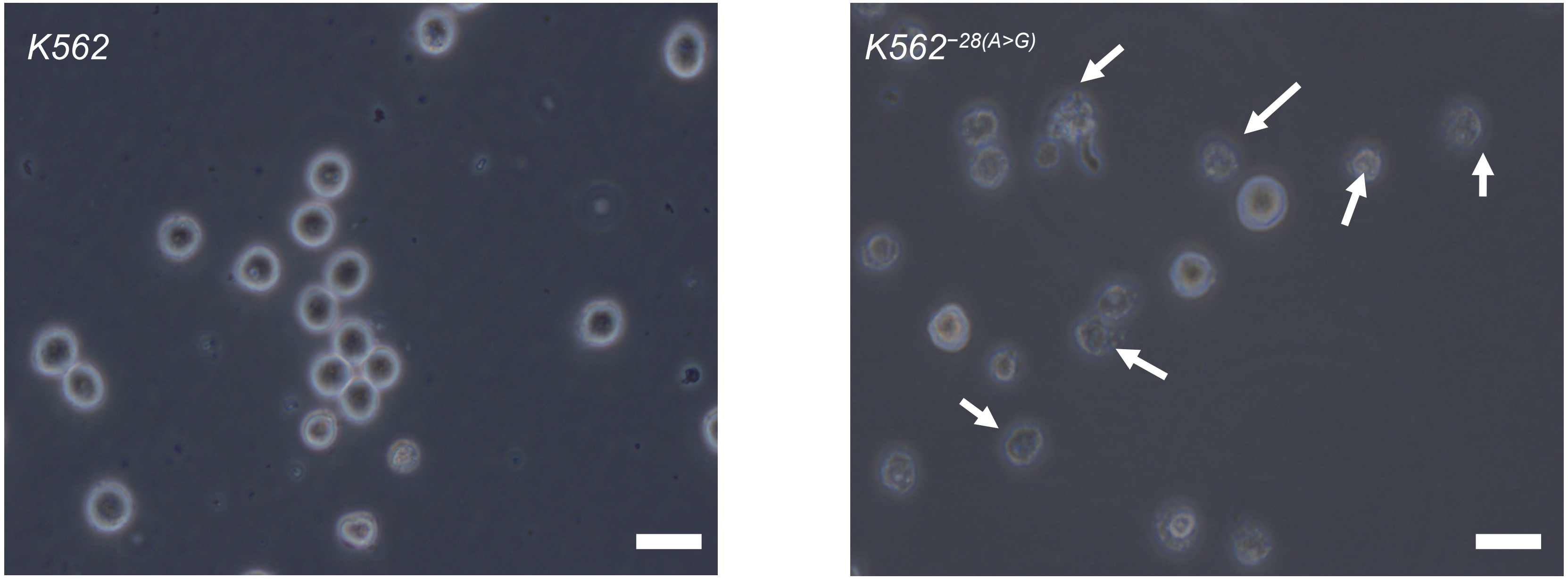

FIGURE S1 | The morphology of K562 and K562−28(A>G) cell lines after prolonged culture. K562 (Left) and K562−28(A>G) (right) cell line cultured in unchanged medium for over 48h. Abnormal cells are indicated with white arrows. Scale bars, 20µm.

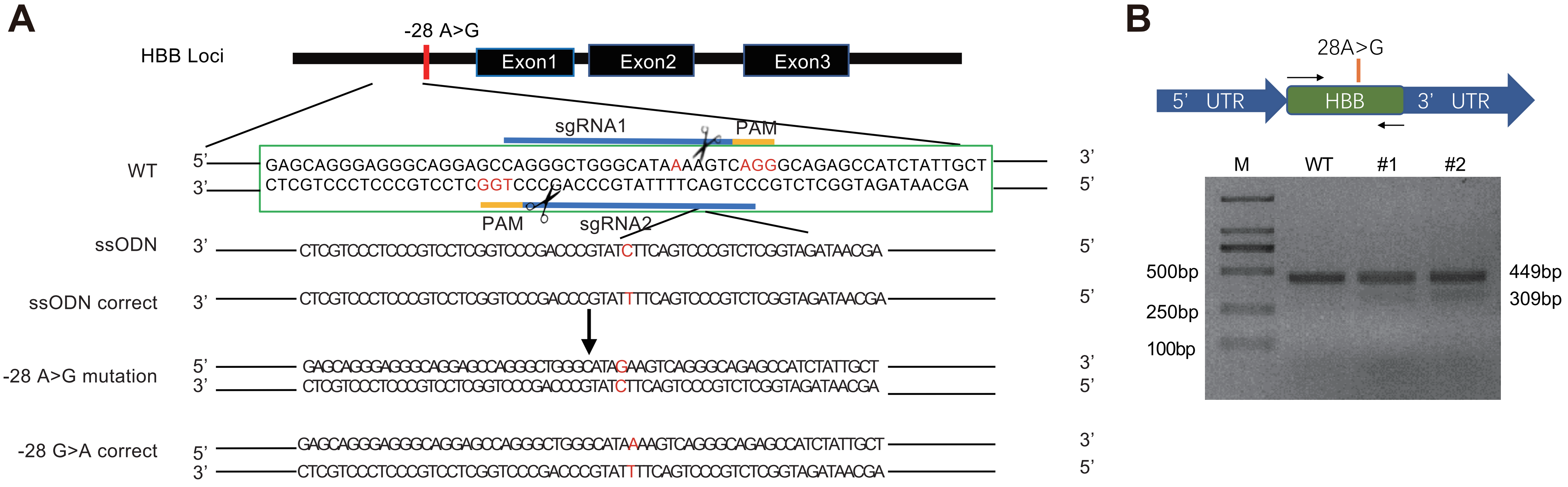

C

		K562 to K562$^{-28(A>G)}$	K562$^{-28(A>G)}$ to K562$^{-28(A>G)cor}$
Single clones Picked		86	60
Sequenced	Indel	7	55
	Hetero	0	2
	Homo	1	1

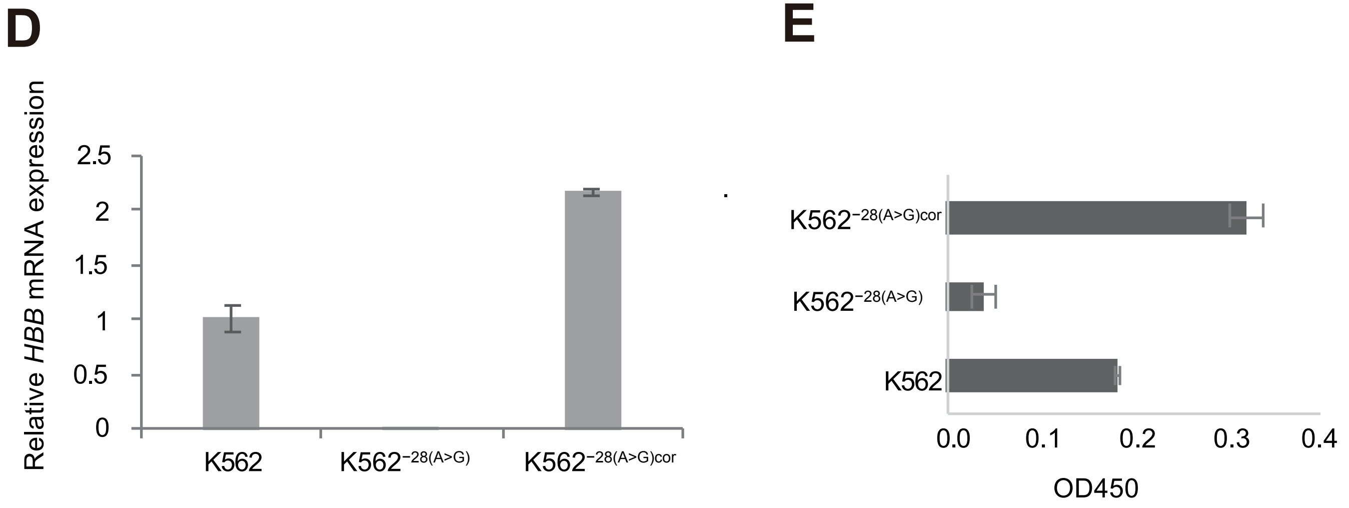

FIGURE S2 | Generation of K562$^{-28(A>G)}$ cell line by asymmetry ssODN combined CRISPR/Cas9.
(A) Schematic of genome editing systems. The region around HBB-28 are targeted with two asymmetric sgRNAs and ssODNs are provided with CRISPR/Cas9 DNA cleavage to generate HBB-28 (A>G) SNP. sgRNA1 and sgRNA2 are complementary to the sense and antisense strands respectively. Mutation site is indicated with red color in the middle of sequence. PAM: protospacer adjacent motif (orange)
(B) Activity assay of gRNA. #1,#2 represent gRNA1 and gRNA2.
(C) A summary of editing efficiency of isogenic cell lines. For mutation, a total of 86 single cell clones were identified by Sanger sequencing. One of them is homozygous, one contains 338bp deletion and 170bp insertion fragment, other clones contain Indels. For correction, total of 60 single clones were identified by Sanger sequencing .Three single clones were successfully corrected as expected. One is homozygous, the other two single clones are heterozygous.
(D) Identification the expression of HBB in the three cell lines by RT-PCR.
(E) Determination of hemoglobin of isogenic lines by TMB. Three repetitions per sample.

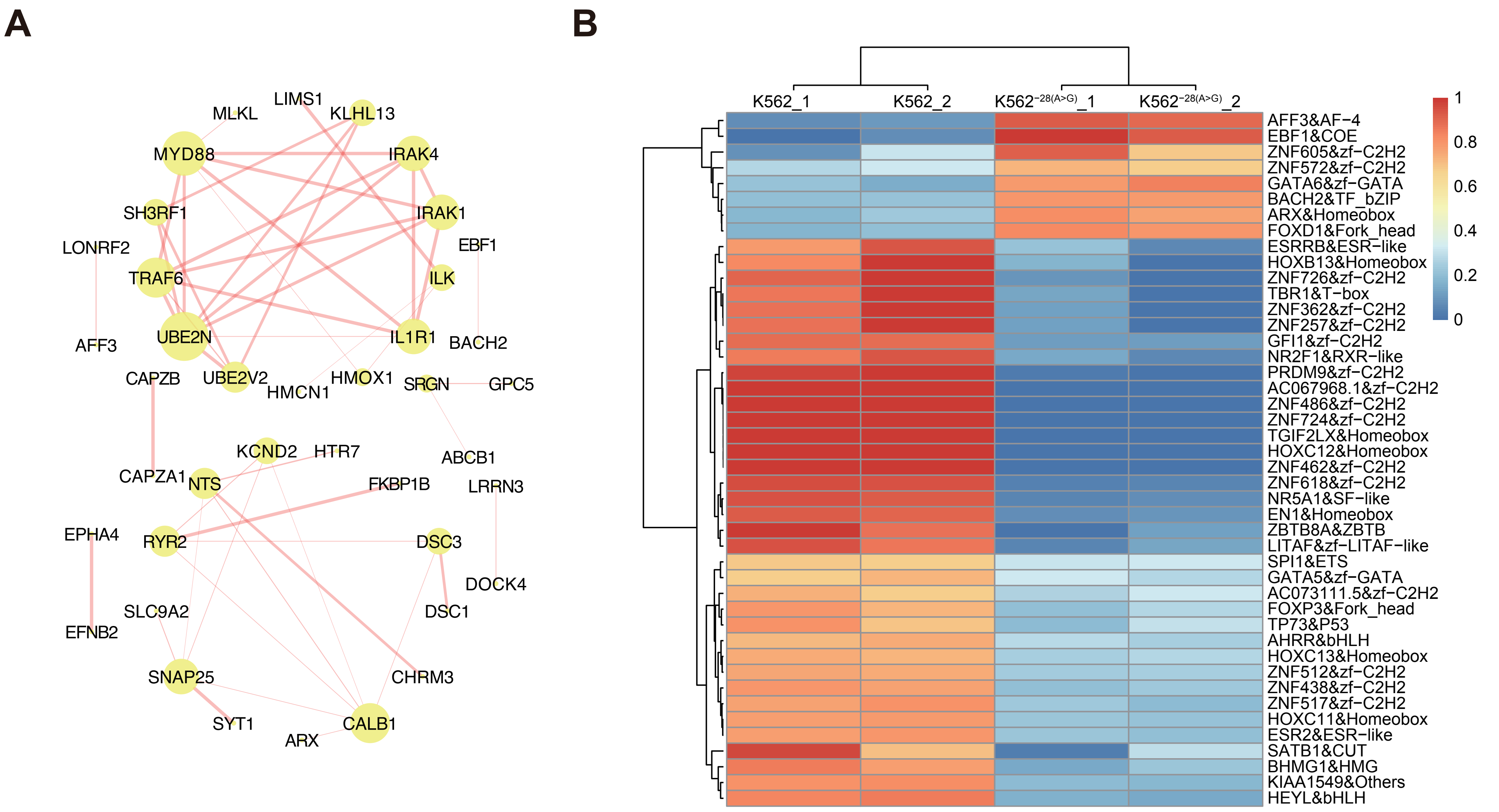

FIGURE S3 | The prediction of protein–protein interactions (PPI) network of hypoxia genes and transcription factors (TFs).
(A) The predicted PPI network of the relationships with 120 upregulated DEGs in K562$^{-28(A>G)}$ were all showed, such as: hypoxia genes (*SRGN*, *HMOX1*, etc.).
The scales of circles are caculated by EdgeCount, the thickness of lines are caculated by combined score.
(B) Prediction of transcription factor in isogenic cell lines pre-induction. K562_1 and K562$^{-28(A>G)}$ _1 in batch 1 and K562_2 and K562$^{-28(A>G)}$ _2 in batch 2 for RNA-Seq;

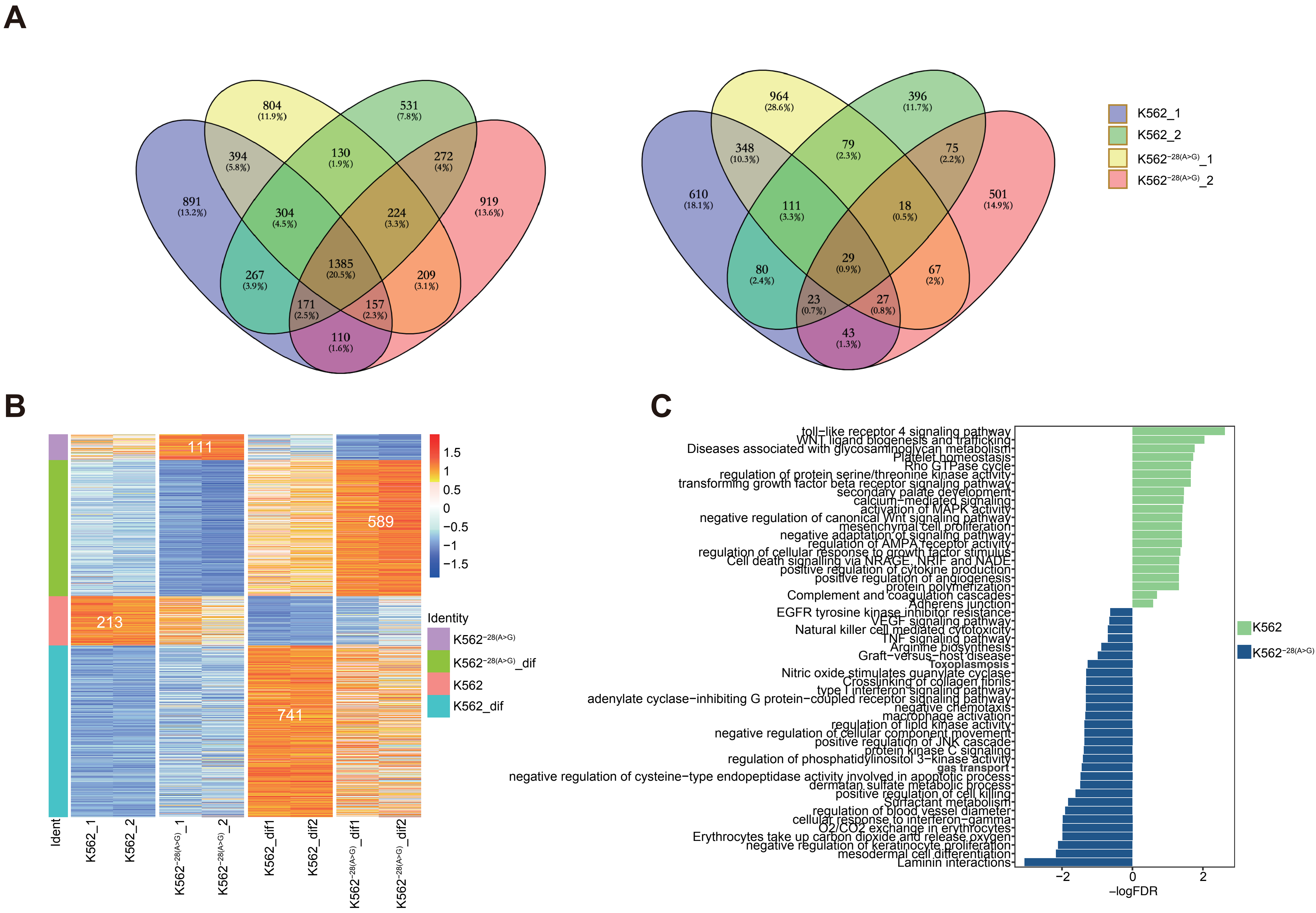

FIGURE S4 | The DEGs and pathways of the K562 and K562$^{-28(A>G)}$ during differentiated processes.
(A) The number of up-regulated (left) and down-regulated (right) genes of two isogenic cells lines in two batches during differentiated processes. (post-induction versus pre-induction, false discovery rate [FDR] < 0.01, log2(fold change [FC]) > 1).
There are 267 upregulated genes in K562 and 209 upregulated genes in K562-28 A>G cell line.1 means batch1, 2 means batch2.
(B) Clustering analyses revealed DEGs between the two isogeneic cell lines during the erythroid differentiation processes.
There were 213, 111, 741 and 589 upregulated genes in K562 (showed in pink), K562-28 A>G(showed in purple), K562_dif (showed in blue) and K562-28 A>G _dif (showed in blue) respectively; dif: differentiation.
(C) Differentially expressed signaling pathways in the two isogenic cell lines during erythroid differentiation processes.

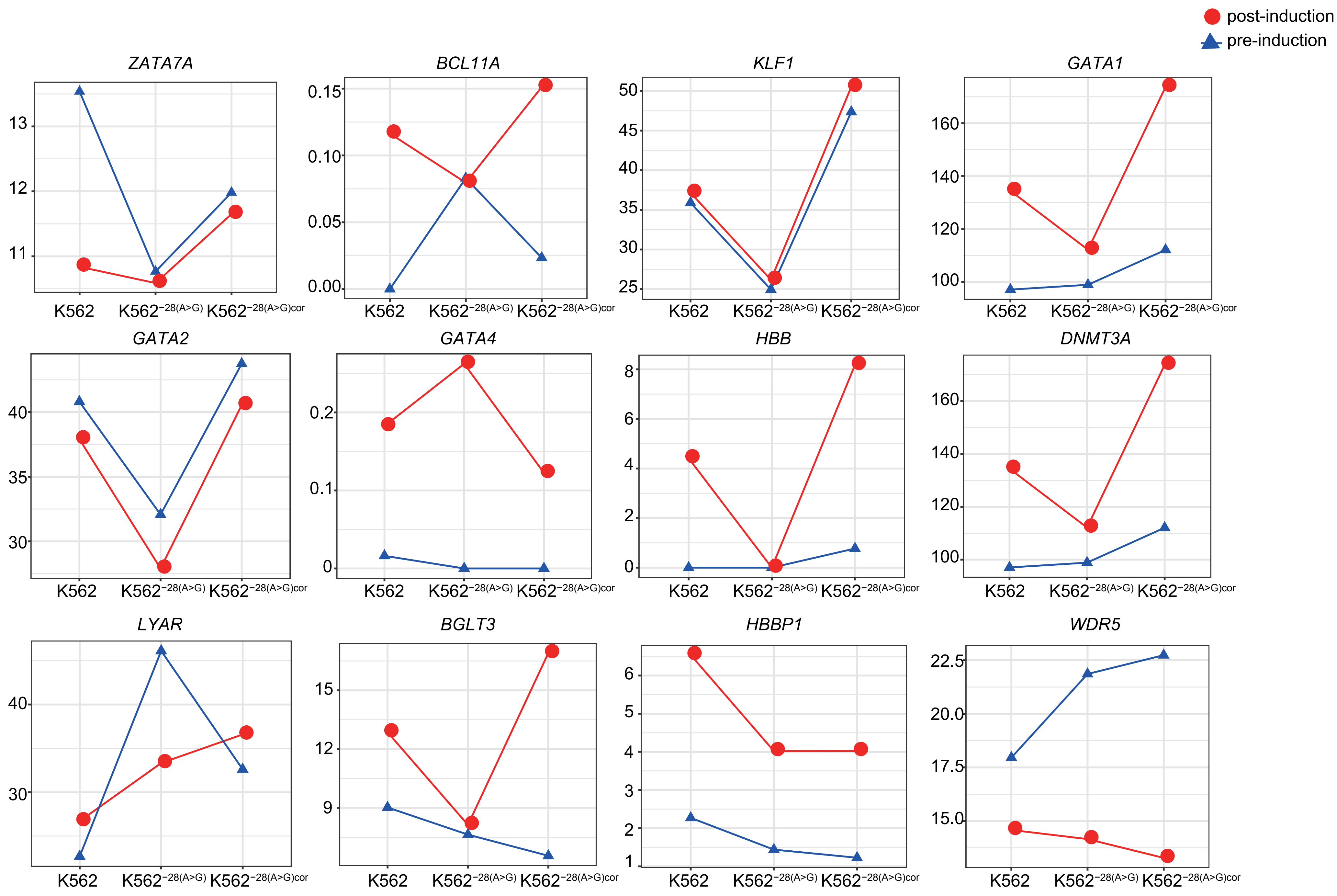

FIGURE S5 | The expression of key transcription factor affecting the globin expression in pre- and post- induction isogenic cell lines. Y axis represent by FPKM. The circles mean post-induction, and triangles mean pre-induction.

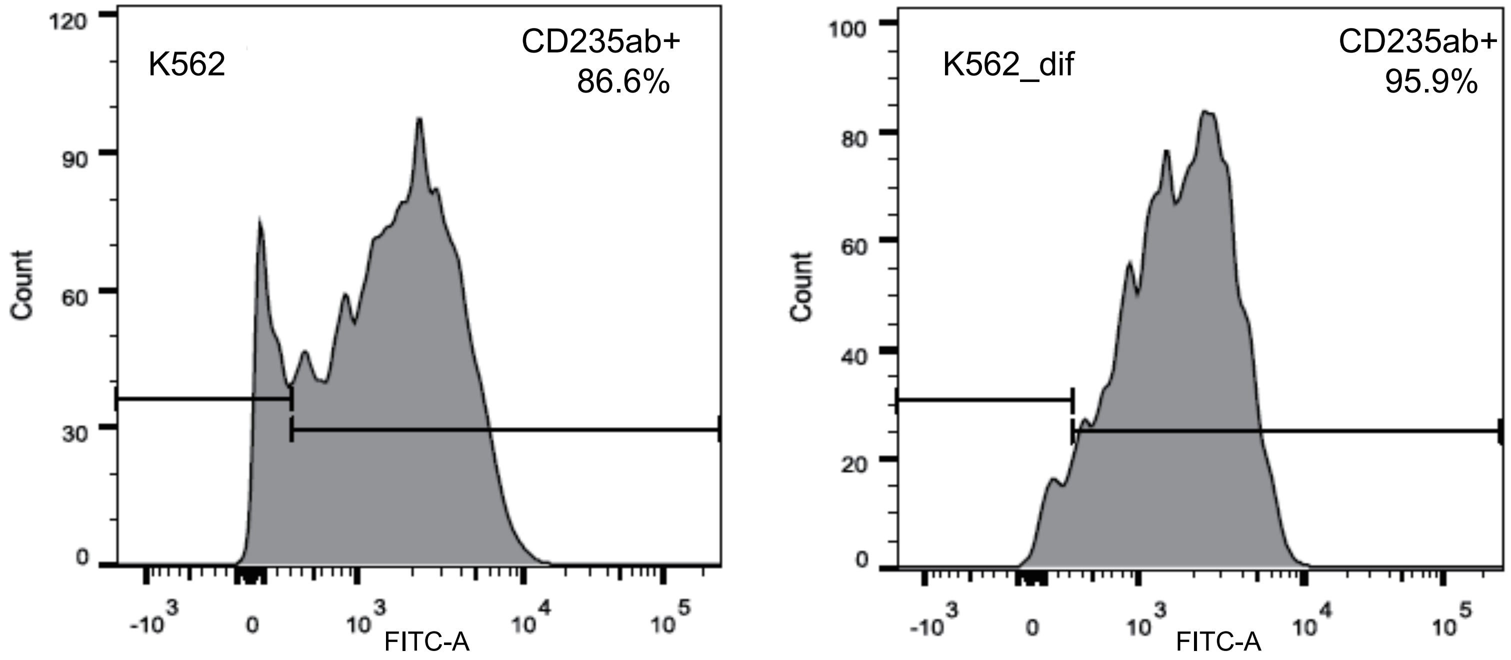

FIGURE S6 | Fluorescence-activated Cell Sorting (FACS) assay for CD235ab+ in pre- and post-induction of K562 cells.
Left: K562 pre-induction; Right: K562 post-induction

Table S1 The key pathways and related genes in K562$^{-28(A>G)}$_dif

**Pathways**	**Genes**	**-Log$_{10}$(*P*-value)**
negative regulation of blood circulation (upregulated)	PDE4D, BIN1, PLN	3.11
negative regulation of leukocyte activation (upregulated)	TBX21, HMOX1, PGLYRP4, SOCS1	2.02
negative regulation of blood coagulation (upregulated)	TFPI, THBS1	1.55
negative regulation of hemostasis (upregulated)	TFPI, THBS1	1.55
negative regulation of hemopoiesis (upregulated)	TBX21, PGLYRP4, SOCS1	1.40
negative regulation of platelet aggregation (downregulated)	ALOX12, SERPINE2, UBASH3B	2.74
Erythrocytes take up carbon dioxide and release oxygen (downregulated)	CA1, AQP1, HBB	2.49
O2/CO2 exchange in erythrocytes (downregulated)	CA1, AQP1, HBB	2.49
negative regulation of platelet activation (downregulated)	ALOX12, SERPINE2, UBASH3B	2.10
regulation of blood pressure (downregulated)	NOS2, PCSK5, ACTA2, P2RX1, ENPEP, AVPR1A, CYP4F2, SLC4A5, SUCNR1, HBB	2.07

Table S2: The expression of mitochondria genes in three isogenic cell lines

TAIR_ID	SYMBOL	Expression in K562	Expression in K562$^{-28(A>G)}$	Expression in K562$^{-28(A>G)cor}$
ENSG00000198712.1	MT-CO2	30692.64844	36744.03516	30770.25977
ENSG00000198727.2	MT-CYB	6165.318359	7140.681641	5964.253418
ENSG00000198763.3	MT-ND2	10497.41895	11490.30371	11902.92871
ENSG00000198804.2	MT-CO1	24538.55078	25426.05859	20618.97852
ENSG00000198840.2	MT-ND3	5910.532227	6203.662598	4681.167969
ENSG00000198886.2	MT-ND4	26198.11133	26931.85742	23103.125
ENSG00000198888.2	MT-ND1	12149.9541	15624.04492	11654.20117
ENSG00000198899.2	MT-ATP6	6574.290527	8397.078125	6698.809082
ENSG00000198938.2	MT-CO3	22358.1543	27259.70703	20360.51367
ENSG00000210082.2	MT-RNR2	19094.99414	14788.95996	20154.99023
ENSG00000210140.1	MT-TC	7179.018066	6783.394531	5114.338867
ENSG00000210144.1	MT-TY	5791.18457	5859.809082	4686.176758
ENSG00000212907.2	MT-ND4L	26618.31641	26450.43555	22554.38477
ENSG00000225630.1	MTND2P28	5174.062012	5560.36377	4965.740234
ENSG00000248527.1	MTATP6P1	6778.824707	8012.343262	5417.968262

The expression of related genes are showed by FPKM.
